# Smoking-related knowledge, attitudes, and behaviors among Alaska Native people: a population-based study

**DOI:** 10.3402/ijch.v72i0.21141

**Published:** 2013-08-05

**Authors:** Kristen Rohde, Myde Boles, Chris J. Bushore, Barbara A. Pizacani, Julie E. Maher, Erin Peterson

**Affiliations:** 1 Program Design and Evaluation Services, Multnomah County Health Department and Oregon Public Health Division, Portland, OR, USA; 2 Tobacco Prevention and Control Program, Alaska State Department of Health and Social Services, Anchorage, AK, USA

**Keywords:** smoking, smoking cessation, Alaska Native people, disparities, indigenous populations

## Abstract

**Background:**

Several studies have shown that Alaska Native people have higher smoking prevalence than non-Natives. However, no population-based studies have explored whether smoking-related knowledge, attitudes, and behaviors also differ among Alaska Native people and non-Natives.

**Objective:**

We compared current smoking prevalence and smoking-related knowledge, attitudes, and behavior of Alaska Native adults living in the state of Alaska with non-Natives.

**Methods:**

We used Alaska Behavioral Risk Factor Surveillance System data for 1996 to 2010 to compare smoking prevalence, consumption, and cessation- and second-hand smoke-related knowledge, attitudes, and behaviors among self-identified Alaska Native people and non-Natives.

**Results:**

Current smoking prevalence was 41% (95% CI: 37.9%–44.4%) among Alaska Native people compared with 17.1% (95% CI: 15.9%–18.4%) among non-Natives. Among current every day smokers, Alaska Natives were much more likely to smoke less than 10 cigarettes per day (OR=5.0, 95% CI: 2.6–9.6) than non-Natives. Compared with non-Native smokers, Alaska Native smokers were as likely to have made a past year quit attempt (OR=1.4, 95% CI: 0.9–2.1), but the attempt was less likely to be successful (OR=0.5, 95% CI: 0.2–0.9). Among current smokers, Alaska Natives were more likely to believe second-hand smoke (SHS) was very harmful (OR=4.5, 95% CI: 2.8–7.2), to believe that smoking should not be allowed in indoor work areas (OR=1.9, 95% CI: 1.1–3.1) or in restaurants (OR=4.2, 95% CI: 2.5–6.9), to have a home smoking ban (OR=2.5, 95% CI: 1.6–3.9), and to have no home exposure to SHS in the past 30 days (OR=2.3, 95% CI: 1.5–3.6) than non-Natives.

**Conclusion:**

Although a disparity in current smoking exists, Alaska Native people have smoking-related knowledge, attitudes, and behaviors that are encouraging for reducing the burden of smoking in this population. Programs should support efforts to promote cessation, prevent relapse, and establish smoke-free environments.

Cigarette smoking is common among Alaska Native people: 41% of Alaska Native adults living in the state of Alaska are current smokers (1), compared with 19% in the US adult population (2). Studies of the disparity in adult smoking among racial and ethnic groups living in the United States typically combine Alaska Native and American Indian populations together (3, 4). To our knowledge, only one published study has examined population-based data on the prevalence of tobacco use among all Alaska Native adults living in the state of Alaska (5). Other studies of tobacco use among Alaska Native people have been focused on a subset of the Alaska Native population, such as pregnant women (6, 7), youth (8), those with children in the home (9), or those in specific areas of the state (10–12).

The primary purpose of this study is to provide current population-based estimates of smoking prevalence among Alaska Native people living in Alaska and augment these data with a broad array of new information on smoking-related knowledge, attitudes, and behaviors. We are unaware of any population-based studies that have assessed this type of information in the state-wide Alaska Native population. It is important to assess knowledge, attitudes, and behaviors surrounding smoking in order to develop effective, culturally tailored interventions that are acceptable to Alaska Native people.

## Method

### Population

Alaska Native people refers to the original inhabitants of the land that is now the state of Alaska. There are more than 120,000 Alaska Native people who currently live in Alaska, and they comprise about 17% of the state's 720,000 residents.

### Data source

We report on data from the Alaska Behavioral Risk Factor Surveillance System (BRFSS) from 1996 to 2010. The Alaska BRFSS is part of the national BRFSS, and it is a population-based, random-digit-dialed, cross-sectional survey stratified on geographic region. Eligible participants are non-institutionalized (i.e. nursing homes, dormitories), aged 18 years or over, who speak English (13). In 1996–2010, the Council of American Survey Research Organizations (CASRO) response rate ranged from 47.7% (year 2000) to 67.5% (year 2005).

Alaska presently conducts 2 BRFSS surveys: the standard BRFSS and a supplemental Alaska BRFSS, which contains many tobacco questions adapted from the Center for Disease Control and Prevention's (CDC's) Adult Tobacco Survey. Both surveys are conducted throughout the year, and separate samples are drawn using the same methodology. At present, approximately 210 Alaska adults are interviewed each month for the standard BRFSS to reach an annual sample size of 2,500; the same number of adults are interviewed for the supplemental BRFSS, for a total of roughly 5,000 survey respondents per year for both surveys. The sample size varied each year, ranging from a low of 1,536 in 1996 to a high of 5,755 in 2005. When possible, we used a data set combining the standard and supplemental BRFSS surveys to provide the estimates contained in this report. In cases where questions appeared on only one of the surveys, we used that particular data set.

### Study measures

#### Demographic measures

We asked all respondents to identify their race, ethnicity, age, gender, highest level of formal education achieved, and whether or not children were present in the home. Regarding race and ethnicity, the survey included a question about whether participants were Hispanic or Latino and a separate question about race. For participants who reported more than one race, we also asked about primary race, “Which one of these groups would you say best represents your race?” For this study, the Alaska Native category includes respondents who reported “Alaska Native/American Indian” as their primary or only race group, and the non-Native category includes all other respondents.

#### Smoking status and quit ratio

We asked all respondents whether they had smoked 100 cigarettes in their lifetimes. For those that responded “yes” (ever smokers) we then asked if they now smoke “every day, some days, or not at all.” Those who responded “every day” or “some days” are considered current smokers. Former smokers are those who had smoked 100 cigarettes, but answered that they now smoke “not at all.” This is a standard smoking measure used in several population-based surveys (14, 15). We also assessed the quit ratio, defined as the proportion of former smokers among ever smokers.

#### Consumption

We asked current smokers “On days when you smoked during the past 30 days, about how many cigarettes did you smoke a day?” We then examined current some day and every day smokers who report smoking less than 10 cigarettes per day.

#### Stage of change

We defined the proportion of current smokers who want to quit smoking by those who answered “yes” to the question, “Would you like to quit smoking?” For those who responded “yes” we then asked if they were seriously considering quitting smoking in the next 6 months. If they responded “yes,” we then asked if they were considering quitting in the next 30 days. Those considering quitting in the next 30 days were considered in the preparation stage of the Stage of Change model (16).

#### Advised to quit

We defined the proportion of smokers advised to quit by a health professional by those who answered “yes” to the question, “In the past 12 months, has a doctor, nurse, or other health professional advised you to quit smoking?” We asked this question only of those respondents who had seen a health professional for care in the past 12 months.

#### Awareness of tobacco quit line

We asked all respondents if they were aware of the Alaska Tobacco Quit Line, a free telephone service that can help people quit smoking. We assessed the proportion of current smokers aware of the service.

#### Past year quit behavior

We combined former smokers who quit 1 year ago or less with all current smokers to examine patterns in past year quit attempts and success. We placed respondents into 3 categories: persons with no quit attempt in the past year, persons with an unsuccessful quit attempt in the past year, and persons with a successful quit attempt in the past year. To examine quit attempts (whether eventually successful or not), we compared the first category to the latter 2 categories combined. To examine quit success, we compared the latter 2 categories to each other.

#### Knowledge of the danger of second-hand smoke (SHS)

We asked all respondents how harmful they thought SHS is to one's health. We dichotomized this measure into very harmful versus all else.

#### Attitudes about clean indoor air policies

We asked all respondents “In indoor work areas do you think that smoking should be allowed in all areas, in some areas, or not allowed at all?” We dichotomized this measure into not allowed at all versus all else. We also asked the same question specifically about restaurants. This variable was also dichotomized into not allowed at all versus all else.

#### Smoking in the home

We asked all respondents, “Which statement best describes the rules about smoking inside your home: smoking is not allowed anywhere inside your home, smoking is allowed in some places or at some times, smoking is allowed anywhere inside your home?” We dichotomized this measure into smoking is not allowed anywhere inside home versus all else.

#### Exposure to SHS

We asked all respondents if anyone had smoked inside their home in the previous 30 days. We dichotomized this measure into zero days versus one or more days.

#### Smoking in the workplace

We asked respondents who reported working indoors most of the time about the official smoking policy at their workplace. We dichotomized this measure into smoking is not allowed in any indoor work area versus all else.

### Analyses

We used data from 1996 to 2010 to examine trends in current smoking prevalence among Alaska Native people and non-Natives. For all other comparisons, we used combined data from 2008 to 2010 to provide the most current estimates of smoking-related knowledge, attitudes, and behavior.

For all analyses, we used the 0.05 level of significance and procedures in Stata^®^ that took into account complex sampling design. Data were weighted to adjust for differential sampling rates within each telephone bank and for the number of telephones and adults in the household, and to ensure that the age, gender, and geographic distribution of respondents matched that for all Alaskans based on the Claritas population estimates for a given year (17). We tested for trends during 1996–2010 in current smoking prevalence with logistic regression. Using data from 2008 to 2010, a Pearson chi-square test with Rao and Scott second-order correction was used to determine whether age, gender, education, and presence of children in the home varied by race (Alaska Native people versus non-Native). Also using 2008–2010 data, we tested for associations between race and smoking-related measures using multiple logistic regression models adjusted for age, gender, and education.

## Results

### Demographics

Compared with non-Natives, Alaska Native people tended to be younger (p<0.001), had less formal education (p<0.001), and were more likely to have children living in the home (p<0.001) (Table I).

**Table I:** Characteristics of Alaska Native and non-Native adults, BRFSS 2008–2010 (N =12,948)

	Alaska Native		Non-Native		
			
	N	Percent*	N	Percent*	*p*
*Age*					<0.001
18–24	209	16.8	476	12.1	
25–34	440	24.4	1,431	21.5	
35–44	422	18.2	1,875	19.2	
45–54	552	19.8	2,534	20.7	
55–64	427	12.3	2,304	15.8	
65 and older	247	8.5	1,690	10.7	
*Gender*					0.52
Male	1,054	50.8	4,742	52.0	
Female	1,289	49.2	5,659	48.1	
*Highest education*					<0.001
Less than high school graduate	454	20.8	492	5.4	
High school graduate or GED	1,102	46.1	2,677	27.8	
College 1–3 years	554	23.7	3,196	30.2	
College graduate	225	9.3	4,022	36.5	
Children in the home**					<0.001
No children in home	432	35.7	2,972	54.7	
Children living in the home	587	64.3	1,743	45.3	

* Percent estimates weighted to adjust for sampling design, gender, and age; counts are unweighted.

** Sample size is smaller because this measure was only on the supplemental BRFSS survey.

### Smoking-related indicators

**Fig. 1:**
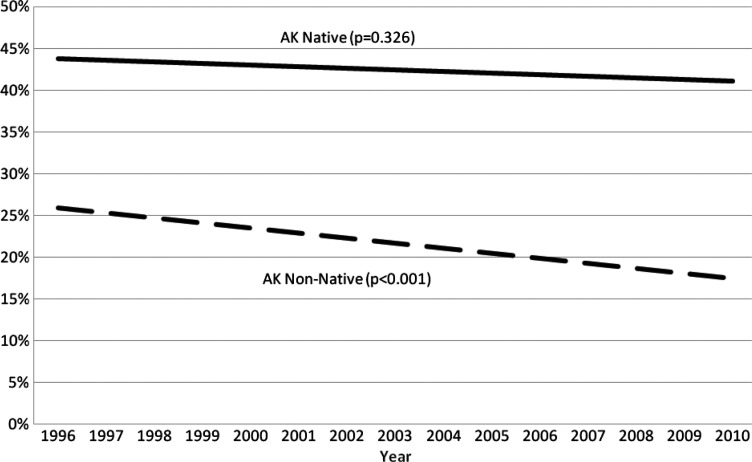
Trends in adult current smoking prevalence, Alaska BRFSS 1996–2010.

The current smoking prevalence among Alaska Native people was more than twice that of non-Natives (Table II). Although smoking prevalence has declined among non-Natives (p<0.001), no significant decline has been observed among Alaska Native people (p=0.33) (Fig. 1).

**Table II:** Prevalence of smoking and related indicators among Alaska Native and non-Native adults, BRFSS 2008–2010

Indicator	Sample size	Prevalence (95% CI)	Adjusted OR (95% CI)
Current smokers			
Alaska Native	2,313	41.1% (37.9–44.4)	2.3 (1.9–2.7)
Non-Native	10,330	17.1% (15.9–18.4)	Referent
Ever smokers			
Alaska Native	2,313	67.7% (64.7–70.5)	2.1 (1.8–2.5)
Non-Native	10,330	44.5% (42.9–46.0)	Referent
Former smokers among ever smokers (quit ratio)			
Alaska Native	1,604	39.3% (35.6–43.1)	0.6 (0.5–0.7)
Non-Native	4,924	61.5% (59.2–63.8)	Referent
Current smokers who smoke every day			
Alaska Native	915	67.7% (62.5–72.5)	0.8 (0.6–1.1)
Non-Native	1,746	70.5% (66.8–74.0)	Referent
Every day smokers who smoke <10 cigarettes per day			
Alaska Native	254	49.7% (37.7–61.8)	5.0 (2.6–9.6)
Non-Native	533	22.6% (16.4–30.4)	Referent
Someday smokers who smoke <10 cigarettes per day			
Alaska Native	99	91.5% (83.2–95.9)	1.2 (0.4–3.2)
Non-Native	204	90.5% (85.0–94.2)	Referent

Note: Counts are unweighted; percentages are weighted. Odds ratios are adjusted for age, gender, and education.

The odds of having ever smoked were significantly higher for Alaska Native people than non-Natives (Table II). Among ever smokers, the odds of being a former smoker (quit ratio) were significantly lower for Alaska Native people. Alaska Native current smokers were as likely to be every day smokers as non-Native current smokers. However, among every day smokers, Alaska Natives had 5 times the odds of smoking less than 10 cigarettes per day. Among some day smokers, Alaska Native and non-Native smokers had the same odds of smoking less than 10 cigarettes per day.

### Cessation-related indicators

Alaska Native smokers had similar odds of wanting to quit smoking and planning to quit smoking in the next 30 days as non-Native smokers (Table III). Alaska Native smokers had decreased odds of being advised by a health professional to quit smoking, although the estimate was not statistically significant (p=0.08). Alaska Native smokers were as likely as non-Native smokers to be aware of the Alaska Tobacco Quit Line. Compared with non-Natives, Alaska Native smokers were as likely to make a quit attempt but were significantly less likely to quit successfully.

**Table III:** Cessation-related indicators among Alaska Native and non-Native adults, BRFSS 2008–2010

Indicator	Sample size	Prevalence (95% CI)	Adjusted OR (95% CI)
Current smokers who want to quit			
Alaska Native	356	76.6% (68.9–82.8)	0.9 (0.5–1.5)
Non-Native	731	77.1% (71.6–81.9)	Referent
Current smokers planning to quit in next 30 days			
Alaska Native	169	46.3% (31.9–61.4)	1.0 (0.5–2.0)
Non-Native	385	47.4% (39.3–55.7)	Referent
Current smokers advised by health professional to quit*			
Alaska Native	198	62.0% (51.9–71.1)	0.6 (0.3–1.1)
Non-Native	513	71.4% (64.4–77.5)	Referent
Current smokers aware of Quit Line			
Alaska Native	388	72.4% (65.0–78.7)	1.1 (0.7–1.7)
Non-Native	762	69.4% (63.4–74.9)	Referent
Past-year smokers who attempted to quit**			
Alaska Native	433	69.4% (62.3–75.7)	1.4 (0.9–2.1)
Non-Native	893	63.7% (58.0–69.0)	Referent
Past-year smokers with successful quit attempt***			
Alaska Native	272	11.9% (7.7–17.9)	0.5 (0.2–0.9)
Non-Native	562	23.6% (18.0–30.2)	Referent

Note: Counts are unweighted; percentages are weighted. Odds ratios are adjusted for age, gender, and education.

* Among current smokers who have seen a health care professional in the past year.

** Quit attempts include those both successful and unsuccessful. Comparison group was past-year smokers with no quit attempt.

*** Among past-year smokers with a quit attempt; Comparison group was past-year smokers with an unsuccessful quit attempt.

### SHS-related indicators

Among smokers and non-smokers, Alaska Native people were significantly more likely to believe that SHS is very harmful (Table IV). Alaska Native smokers and non-smokers were also significantly more likely to agree that smoking should not be allowed at all in indoor work areas and in restaurants.

**Table IV:** Second-hand smoke-related indicators among Alaska Native and non-Native adults, BRFSS 2008–2010

Indicator		Sample size	Prevalence (95% CI)	Adjusted OR (95% CI)
*Believe second-hand smoke is very harmful*				
Current smokers	Alaska Native	391	71.1% (63.6–77.7)	4.5 (2.8–7.2)
	Non-Native	771	35.5% (29.5–42.0)	Referent
Non-smokers	Alaska Native	617	77.3% (72.2–81.7)	1.8 (1.3–2.5)
	Non-Native	3,920	66.1% (63.7–68.5)	Referent
*Believe that smoking should not be allowed in indoor work areas*				
Current smokers	Alaska Native	385	73.5% (65.8–80.0)	1.9 (1.1–3.1)
	Non-Native	741	63.3% (57.3–69.0)	Referent
Non-smokers	Alaska Native	599	91.5% (87.6–94.2)	1.8 (1.1–2.9)
	Non-Native	3,823	88.6% (86.7–90.2)	Referent
*Believe that smoking should not be allowed in restaurants*				
Current smokers	Alaska Native	383	82.9% (77.1–87.4)	4.2 (2.5–6.9)
	Non-Native	755	58.5% (52.2–64.5)	Referent
Non-smokers	Alaska Native	605	92.0% (89.0–94.3)	2.1 (1.4–3.1)
	Non-Native	3,846	85.7% (83.9–87.4)	Referent
*Home smoking ban*				
Current smokers	Alaska Native	388	85.4% (80.6–89.2)	2.5 (1.6–3.9)
	Non-Native	768	68.1% (62.8–73.0)	Referent
Non-smokers	Alaska Native	609	96.6% (94.8–97.8)	2.0 (1.2–3.4)
	Non-Native	3,891	94.9% (93.8–95.9)	Referent
*No home second-hand smoke exposure in past 30 days*				
Current smokers	Alaska Native	389	86.0% (81.0–89.9)	2.3 (1.5–3.6)
	Non-Native	770	70.5% (65.3–75.2)	Referent
Non-smokers	Alaska Native	613	97.4% (95.6–98.4)	1.9 (1.0–3.6)
	Non-Native	3,917	96.6% (95.6–97.4)	Referent
*Workplace smoking ban*				
Current smokers	Alaska Native	273	50.0% (38.7–61.4)	0.6 (0.4–1.1)
	Non-Native	555	67.7% (59.9–74.6)	Referent
Non-smokers	Alaska Native	425	80.8% (75.1–85.4)	0.9 (0.6–1.4)
	Non-Native	2,836	84.4% (81.9–86.6)	Referent

Note: Counts are unweighted; percentages are weighted. Odds ratios are adjusted for age, gender, and education.

Alaska Native smokers and non-smokers were significantly more likely to have both a home smoking ban and no home exposure to SHS in the past 30 days as compared to non-Native smokers and non-smokers (Table IV). In the workplace, Alaska Native smokers were less likely to have a smoking ban, although this association did not reach statistical significance (p=0.12). Among non-smokers, there was no significant difference between Alaska Natives and non-Natives in the likelihood of having a workplace smoking ban.

## Discussion

Although the disparity in smoking among Alaska Native people continues to persist—twice as many Alaska Native people smoke cigarettes compared with non-Natives—our results suggest that Alaska Native people have knowledge, attitudes, and behaviors that could support reductions in the burden of smoking. For example, Alaska Native every day smokers consume fewer cigarettes per day than non-Native smokers, potentially indicating lower addiction levels (18). In addition, they are equally likely to want to quit, to be ready to quit, and to actually attempt to quit as non-Native smokers. However, Alaska Native smokers had half the odds of making a successful quit attempt compared to non-Natives. These findings contradict other evidence that lower addiction levels are associated with lower risks of relapse after a quit attempt (19). Finding ways to address relapse triggers such as stress and depression, which are high in Alaska Native people (20, 21), could be helpful.

In addition, awareness of the Alaska Tobacco Quit Line was high among Alaska Native smokers. In a separate study, Alaska Native users of the Quit Line had high quit rates (22%) and nearly all (90%) reported satisfaction with the service, but the service was underutilized by Alaska Native smokers (22). Further outreach could be done to increase use of the Tobacco Quit Line or to explore alternative cessation interventions in the context of a public health approach.

Taken together, these findings provide important information about how to tailor cessation and relapse prevention efforts in this population. We found it encouraging that a much larger proportion of Alaska Native smokers had knowledge regarding the harms of SHS and very favourable attitudes regarding smoke-free environments in their communities compared with non-Native smokers. Further, Alaska Native smokers were more likely to have home smoking bans. This suggests that public health messages regarding the harms of SHS are reaching the Alaska Native population, and that social norms regarding tobacco may be changing in this population. However, we observed that Alaska Native smokers might be less likely to work in smoke-free environments. Because smoke-free environments support cessation (23, 24), this is an area that should be specifically investigated.

### Limitations

This study has several limitations. First, the BRFSS excludes individuals who live in homes without landline telephones, those who live in institutions, and those who do not speak English. As noted in a recent study of Alaska Native people, 8% of Alaska Native respondents spoke only native languages (25). These Native people would not be captured in the Alaska BRFSS, which is given only in English, and their smoking related knowledge, attitudes, and behavior may be different than those of Alaska Native people who speak English. Second, Alaska Native people might be reluctant to report behaviors and attitudes that others might not find acceptable (particularly over the telephone to a stranger). For example, in another study utilization rates of the Alaska Tobacco Quit Line were lower among Alaska Native smokers compared with non-Native smokers, suggesting that Alaska Natives may be uncomfortable revealing personal information over the telephone (22). Future researchers may want to investigate this potential limitation by comparing results obtained through different data collection strategies.

## Conclusions

Despite the large disparity in current smoking prevalence, Alaska Native people living in Alaska have smoking-related knowledge, attitudes, and behaviors that suggest they want to quit and strongly support smoke-free environments. Public health programs should continue to monitor and explore patterns in quit behaviors including relapse in this population. Additionally, in order to achieve smoking prevalence reductions, efforts should explore culturally appropriate ways to promote cessation and to prevent relapse among Alaska Native smokers, and to build upon the strong preference among Alaska Native people for smoke-free environments.
